# Understanding DNA Epigenetics by Means of Raman/SERS Analysis for Cancer Detection

**DOI:** 10.3390/bios14010041

**Published:** 2024-01-12

**Authors:** Luca David, Anca Onaciu, Valentin Toma, Rareș-Mario Borșa, Cristian Moldovan, Adrian-Bogdan Țigu, Diana Cenariu, Ioan Șimon, Gabriela-Fabiola Știufiuc, Eugen Carasevici, Brîndușa Drăgoi, Ciprian Tomuleasa, Rareș-Ionuț Știufiuc

**Affiliations:** 1 Faculty of Medicine, “Iuliu Hatieganu” University of Medicine and Pharmacy, 400349 Cluj-Napoca, Romania; david.luca@elearn.umfcluj.ro; 2 MedFuture—Research Center for Advanced Medicine, “Iuliu Hatieganu” University of Medicine and Pharmacy, 400349 Cluj-Napoca, Romania; anca.onaciu@umfcluj.ro (A.O.); valentin.toma@umfcluj.ro (V.T.); rares.mari.borsa@elearn.umfcluj.ro (R.-M.B.); moldovan.cristian@umfcluj.ro (C.M.); bogdan.tigu@umfcluj.ro (A.-B.Ț.); diana.cenariu@umfcluj.ro (D.C.); ciprian.tomuleasa@umfcluj.ro (C.T.); 3 Department of Pharmaceutical Physics & Biophysics, “Iuliu Hatieganu” University of Medicine and Pharmacy, 400349 Cluj-Napoca, Romania; 4 Department of Maxillofacial Surgery and Implantology, “Iuliu Hațieganu” University of Medicine and Pharmacy, 400349 Cluj-Napoca, Romania; 5 Department of Surgery, “Iuliu Hatieganu” University of Medicine and Pharmacy, 400349 Cluj-Napoca, Romania; ioan.simon@umfcluj.ro; 6 Faculty of Physics, “Babes Bolyai” University, 400084 Cluj-Napoca, Romania; gabriela.stiufiuc@ubbcluj.ro; 7 Nanotechnology Laboratory, TRANSCEND Research Center, Regional Institute of Oncology, 700483 Iasi, Romania; eugen.carasevici@gmail.com (E.C.); transcendbd@iroiasi.ro (B.D.); 8 Department of Hematology, “Iuliu Hatieganu” University of Medicine and Pharmacy, 400349 Cluj-Napoca, Romania; 9 Department of Hematology, “Ion Chiricuta” Clinical Cancer Center, 400015 Cluj-Napoca, Romania

**Keywords:** DNA, multiple myeloma, epigenetics, Raman-SERS spectroscopy, plasmonic substrates

## Abstract

This study delves into the intricate interaction between DNA and nanosystems, exploring its potential implications for biomedical applications. The focus lies in understanding the adsorption geometry of DNA when in proximity to plasmonic nanoparticles, utilizing ultrasensitive vibrational spectroscopy techniques. Employing a combined Raman-SERS analysis, we conducted an in-depth examination to clarify the molecular geometry of interactions between DNA and silver nanoparticles. Our findings also reveal distinctive spectral features regarding DNA samples due to their distinctive genome stability. To understand the subtle differences occurring between normal and cancerous DNA, their thermal stability was investigated by means of SERS measurement performed before and after a thermal treatment at 94 °C. It was proved that thermal treatment did not affect DNA integrity in the case of normal cells. On the other hand, due to epimutation pattern that characterizes cancerous DNA, variations between spectra recorded before and after heat treatment were observed, suggesting genome instability. These findings highlight the potential of DNA analysis using SERS for cancer detection. They demonstrate the applicability of this approach to overcoming challenges associated with low DNA concentrations (e.g., circulating tumor DNA) that occur in biofluids. In conclusion, this research contributes significant insights into the nanoscale behavior of DNA in the presence of nanosystems.

## 1. Introduction

In the realm of biomedicine, cancer remains a significant challenge, with increasing mortality rates, disease complexity, and specificity, making it the second leading cause of death in the European Union [1]. In 2018, Europe saw an estimated 3.91 million new cancer cases (excluding non-melanoma skin cancer) and 1.93 million cancer-related deaths [1]. While treating cancer remains complex due to its adaptive nature, research offers hope in early detection, enabling earlier and more proactive treatment approaches.

Hematological malignancies are among the common types of cancers, and of all these abnormal proliferations, multiple myeloma (MM) accounts for 15% of reported cases worldwide, representing the second most prevalent [2]. In 2020, according to Globocan statistics, MM presented an incidence of 180,000 new cases and about 117,000 deaths.

MM cells’ genome is characterized by extensive rearrangements caused by DNA damage. Although DNA damage mechanisms are not yet completely elucidated, there are some presuppositions in the direction of DNA replication because of cellular stress [3]. DNA replication plays a fundamental role in cell proliferation and in maintaining genome integrity. Stressful events involved in MM cell development affect DNA repair pathways and lead to genomic instability [4].

Epigenetic mechanisms are crucial for normal development and the maintenance of tissue-specific gene expression patterns. Disruption of these processes can lead to functional changes and differentiation into tumor cells. Tumor DNA is characterized by global epigenetic modifications that cooperate with genetic alterations [5]. Recent studies suggest various epimutations specific to malignant cells, encompassing specific changes in DNA methylation, histone structure, nucleosome positioning, and non-coding RNA expression (especially microRNAs) [6,7].

Among these mechanisms, DNA methylation is studied in detail and serves as a mechanism for modifying gene expression by influencing chromatin architecture. This process involves adding a methyl group to the C5 position of the pyrimidine ring of cytosine, generating 5-methylcytosine (5 mC) [8]. While CpG methylation, occurring in the context of CpG dinucleotides, is well known for gene inactivation, non-CpG methylation also plays a role in regulating tissue-specific gene expression. This regulation is mediated by a group of enzymes, DNA methyltransferases, which deactivate gene transcription [9,10]. Aberrant changes in DNA methylation were among the first recognized epimutations in the mechanism of malignant cell formation [11]. Furthermore, hypomethylation of oncogene promoters can increase their expression [12], leading to genomic instability [13,14], while abnormal hypermethylation of specific sequences in cancer cells can also contribute to genomic instability by inactivating genes involved in cell cycle regulation and DNA repair [15].

From a medical perspective, the treatment begins with two major considerations: firstly, the quantification of disturbance within our body caused by the emergence, growth, and proliferation of cancer cells, and secondly, the development of innovative detection and treatment methods using previously established technologies for the different cancer types. Early cancer diagnosis significantly improves the chances for successful treatment, increasing the survival rate at one and five years [16]. The most related MM diagnosis methods are PCR (polymerase chain reaction) and NGS (next-generation sequencing), but unfortunately, they present bad time management.

Therefore, with the aim of overcoming the mentioned obstacles above, our goal is the implementation of a new, cheaper, time-efficient method possessing high sensitivity and specificity. Ultrasensitive vibrational methods such as Raman and surface-enhanced Raman spectroscopy (SERS) could represent a valuable response to these demands. Although many studies presenting valuable findings have been conducted [17,18], these methods are at the beginning in the field of DNA analysis, offering novel and promising approaches for advancing diagnosis.

Raman spectroscopy is employed for the analysis of proteins, DNA, and chromosomes, providing insights into both the chemical composition and secondary structures within these molecules. However, a significant drawback of this method is its demand for a high DNA concentration in the solution. Consequently, it proves challenging to use solutions extracted from biological fluids, particularly those from cancer patients, due to their low DNA concentration.

An alternative approach to detect nucleic acids using label-free sequences is SERS. This method relies on the attachment of a suitable Raman-active molecule to a metal substrate. This metal substrate can be either electrodes, vapor-deposited films, or nanoparticles. While the choice of metal for enhancement can vary, the most used are gold and silver, the last one typically providing the most substantial signal enhancements [19]. Raman scattering can be boosted by up to 10^14^ times at specific sites referred to as “hot spots”, highlighting the potential of SERS spectroscopy for single-molecule detection [20,21,22].

Both methods are non-invasive and do not require additional labels or staining procedures, therefore preserving the sample integrity. Moreover, they allow real-time monitoring of molecular changes, providing insights into the progression of diseases or the response to treatments. Considering these numerous advantages, our research group has achieved remarkable results based on the Raman/SERS technique’s ability to distinguish between normal and diseased samples at the molecular level using multivariate analysis tools [23,24,25]. Sample preparation represents a step of ultimate importance in our protocols due to the fact that good-quality probes lead to accurate results.

In our study, we have evaluated various methods to prepare DNA samples extracted from different cell lines for spectroscopic analysis without using aggregating agents. The selection of the proper method for the spectral analysis of DNA samples that can support the development of potential diagnosis strategies is of major importance. To achieve this goal, understanding the interaction between DNA and nanoparticles is necessary, considering the genomic instability of tumors due to epimutations.

## 2. Materials and Methods

### 2.1. Cell Culturing

LX2—normal hepatic cell line, maintained in DMEM 4.5 g/L glucose medium supplemented with 5% Fetal Bovine Serum, 1% glutamine, and 1% Penicillin/Streptomycin; CCD1137Sk—normal epithelial cell line, maintained in IMDM medium supplemented with 10% Fetal Bovine Serum, 1% glutamine, and 1% Penicillin/Streptomycin; MM1S—multiple myeloma cell line, maintained in RPMI1640 medium supplemented with 10% Fetal Bovine Serum, 1% glutamine and 1% Penicillin/Streptomycin; U266—multiple myeloma cell line, maintained in RPMI1640 medium supplemented 15% Fetal Bovine Serum, 1% glutamine, and 1% Penicillin/Streptomycin. All the cell lines were purchased from ATCC and maintained in a humidified chamber, in sterile conditions, at 37 °C and with 5% CO_2_. All the reagents used at this step were purchased from Gibco (Grand Island, NY, USA). Before being used in experiments, all cell lines underwent a Mycoplasma test. Mycoplasma contamination was verified using MycoAler PLUS Mycoplasma Detection Kit (Lonza Bioscience, Basel, Switzerland), which is based on a luminescent assay. The samples were read using the TECAN SPARK10M Spectrophotometer (Tecan, Männedorf, Switzerland) using the bioluminescence application available on this machine.

### 2.2. DNA Extraction

The four adherent cell lines underwent a detaching step using Trypsin + EDTA solution (Grand Island, NY, USA) and were incubated for 2 min at 37 °C. The cells were counted using an automated cell counter (EVE cell counting, NanoEntek, Hwaseong, Republic of Korea) and aliquoted in 1.5 mL tubes at a final volume of 200 µL of Phosphate Buffer Saline 1X (Grand Island, NY, USA).

In the beginning, the cell samples were washed three times with PBS 1× at 161× *g* for 5 min at room temperature. The cell pellet was resuspended in 200 µL of PBS 1× and stored at −80 °C for at least 24 h. DNA extraction was performed by following the PureLink DNA Extraction and Purification Kit protocol (Invitrogen, Carlsbad, CA, USA).

The DNA concentration was determined with the aid of a Nanodrop 2000c system (Thermo Fisher Scientific, Waltham, MA, USA).

Finally, all the samples were prepared at a concentration of 100 ng/µL and stored at −80 °C until Raman and SERS analyses.

### 2.3. DNA Methylation Analysis

DNA methylation analysis was performed by using the Global DNA Methylation Assay Kit (Abcam, Waltham, MA, USA). We used a DNA concentration of 100 ng/µL. The analysis was performed according to the protocol provided by the manufacturer. The absorbances were measured at an excitation wavelength of 450 nm using a multimode microplate reader (Tecan Spark 10M, Tecan, Wien, Austria). The analysis was performed in biological duplicates.

### 2.4. DNA Base Preparation

Pure DNA bases (adenine, guanine, thymine, cytosine) were purchased from the producers (Sigma Aldrich, Taufkirchen, Germany). Solutions containing these bases, having a concentration of 100 ng/µL, were prepared by dissolving the powders in RNase/DNase-free ultrapure water.

### 2.5. Colloidal Silver Nanoparticle Synthesis

Colloidal silver nanoparticles were obtained according to Leopold and Lendl method [26] using a reduction process of silver nitrate with hydroxylamine hydrochloride. The as-synthesized nanoparticles were subjected to a tangential flow filtration (TFF) procedure to concentrate and purify them, in a similar manner to that reported in previous studies, allowing a high reproducibility degree of the SERS spectra recorded on different biofluids [23,25,27]. After this, the 10× concentrated silver nanoparticles were appropriate for use as solid SERS substrates following the method developed by our group a couple of years ago [28]. The silver nanoparticles were characterized using UV-Vis spectroscopy (Figure S1) and transmission electron microscopy (Figure S2).

### 2.6. Raman Spectroscopy Sample Preparation and Measurements

Raman analysis involved the deposition of the DNA samples of 100 ng/µL on aluminum foil as a port probe previously cleaned and allowed to dry at room temperature prior to Raman spectra acquisition. The same method was applied in the case of all the solutions of DNA bases.

Raman measurements were performed on a Renishaw™ inVia Reflex Raman confocal multilaser spectrometer (Renishaw plc, Gloucestershire, UK) using a laser excitation of 532 (for DNA samples) or 785 nm (for DNA bases). The laser power measured on the sample surface was 113 mW (for the 532 nm laser) and 65 mW for the NIR laser. For each DNA sample, 2 maps of 10 acquisition points and 40 s integration time were recorded. In the case of DNA bases, a single map of 10 acquisition points was recorded, the final spectrum being the mean of the individual spectra. All the spectra were measured at a maximum 30 µm distance from the edge using a 50× objective.

### 2.7. SERS Sample Preparation and Measurements

Prior to DNA sample measurements, the substrate was investigated before and after heating treatment by means of SERS. In the first case, silver nanoparticles were poured on CaF_2_ Raman-grade glass and then allowed to dry at room temperature. In the second case, silver nanoparticles were poured on CaF2 Raman-grade glass and heated at 94 °C for 4 min in a thermoblock (Eppendorf ThermoMixer C). Both samples were then measured.

DNA samples were mixed with silver nanoparticles at a 1:1 volume ratio and incubated for 1 h at room temperature. Then, the mixtures were deposited on CaF_2_ Raman-grade glass. Two different temperature conditions were applied before the SERS analysis. For the first round of measurements, the mixtures were allowed to dry at room temperature before SERS spectra recording. The second round of measurements involved the incubation of the mixture at 94 °C for 4 min in a thermoblock followed by the recording of SERS. The same method was applied in the case of the four DNA bases.

SERS spectra were recorded on a Renishaw™ *inVia* Reflex Raman confocal multilaser spectrometer (Renishaw plc, Gloucestershire, UK) using a laser of 785 nm excitation and 1.95 mW power. All the measurements were performed at a maximum 30 µm distance from the edge using the 50× objective. For each sample, 2 maps of 50 acquisition points using an integration time of 10 s were recorded. The final spectrum represents the mean of the individual spectra.

## 3. Results

### 3.1. DNA Methylation Assay

Before proceeding to cell culture experiments, all the cell lines were tested for Mycoplasma, and the results were negative, as presented in Table S1.

The DNA methylation results are presented in Table 1.

It is notable that the DNA methylation values present a higher level in the case of normal cells as compared to MM cell lines. This assay’s results reflect a general hypomethylation of DNA samples extracted from the cancer cells included in this study, as compared to normal ones. The hypomethylation is present in the case of both cancer cell lines, being more pronounced for U266 cells (0.160%) as compared to MM1S (0.268%). DNA hypomethylation can have harmful effects, identified in all types of cancer: genomic instability (due to chromosomal instability and increased mutation rates), activation of oncogenes, reactivation of transposable elements, loss of imprinting, and altered immune response. From a structural point of view, DNA hypomethylation in cancer DNA can impact the overall stability of the DNA molecule.

### 3.2. Raman Analysis

**Figure 1:**
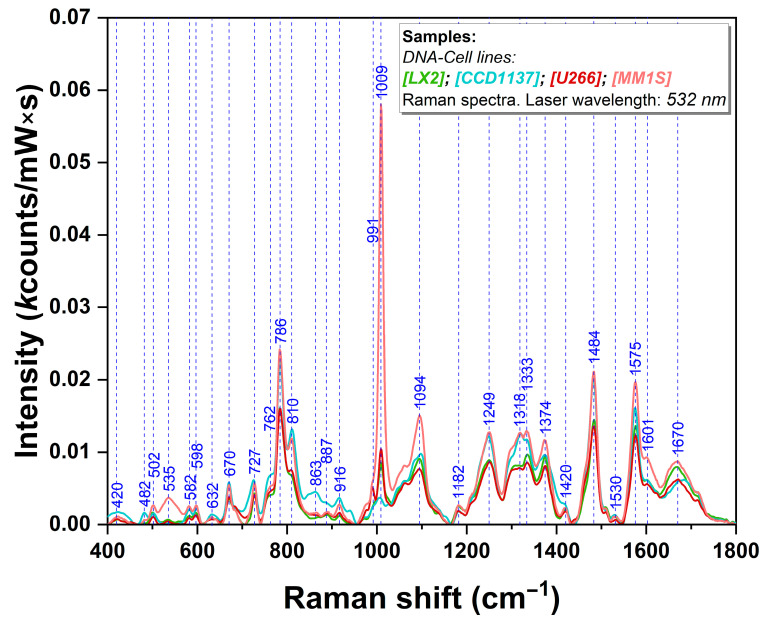
Raman spectra of DNA samples isolated from normal (LX2—green; CCD1137Sk—blue) and cancer cells (U266—red; MM1S—pink). The spectra were recorded using a 532 nm excitation laser.

Raman measurements performed on double-stranded (ds) DNA samples isolated from normal (LX2&CCD1137) and MM cells (U266&MM1S) are presented in Figure 1. One can notice that in the case of all DNA samples, there are some high-intensity vibrational bands located at 670, 727, 786, 1009, 1094, 1249, 1333, 1374, 1484, and 1575 cm^−1^.

Among these bands, one can find out those assigned to the breathing modes of the DNA bases: 670 cm^−1^—ring breathing mode of thymine (T) and guanine (G); 727 cm^−1^—ring breathing mode of adenine (A); and 786 cm^−1^—ring breathing mode of cytosine (C) [29]. Other bands can be assigned to different vibrational modes of the four bases (1249 cm^−1^—T; 1333 cm^−1^—A; 1374 cm^−1^—T, A, G; 1484 cm^−1^—A, G; 1575 cm^−1^—A, G) or to vibrational modes of the backbone (bk): 786, 810, and 1094 cm^−1^ [21]. The latter is the most intense band assigned to bk vibrations, and its intensity can represent a very useful tool for studying the interaction geometry of dsDNA with the plasmonic nanoparticles in the case of Raman/SERS analysis of DNA samples, as previously shown by Onaciu et al. [27].

**Figure 2:**
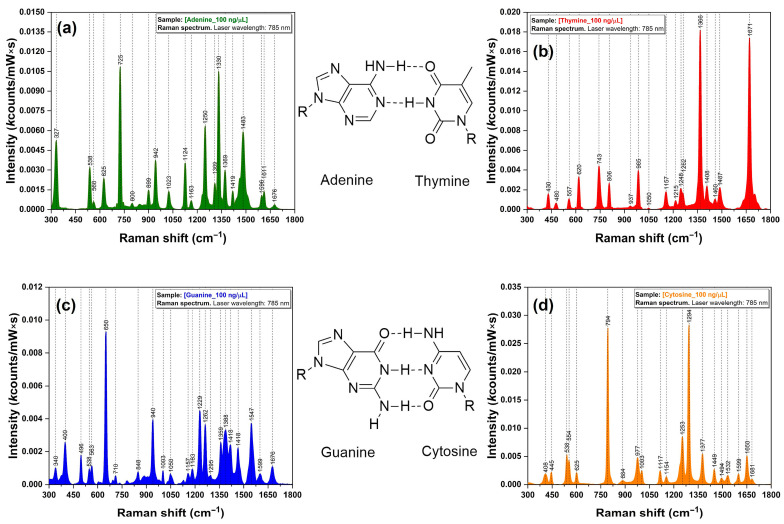
Raman spectra of DNA bases (**a**) A (green), (**b**) T (red), (**c**) G (blue), and (**d**) C (orange) recorded with a 785 nm excitation laser. The geometry of interaction occurring between the complementary bases is also highlighted in the figure.

For a proper band assignment of these vibrational bands, a Raman analysis of the four DNA bases (A, T, C, and G) has been performed. The spectra are presented in Figure 2. The formation of the two or three hydrogen bonds between the complementary bases (A/T, C/G) is also highlighted in the figure.

In the case of A, the spectrum exhibits a very intense band associated with the breathing mode (725 cm^−1^) and several intense bands in the 1200–1700 cm^−1^ region. The same behavior is observed for C and G with the observation that in their cases the breathing modes are located at 794 cm^−1^ (C) and 650 cm^−1^ (G). T is the only base that does not “obey” this spectral pattern, with the two main bands occurring at 1366 and 1671 cm^−1^. These bands were assigned to C-H and N-H in-plane bending and to C=O stretching, respectively [21].

### 3.3. SERS Analysis

**Figure 3:**
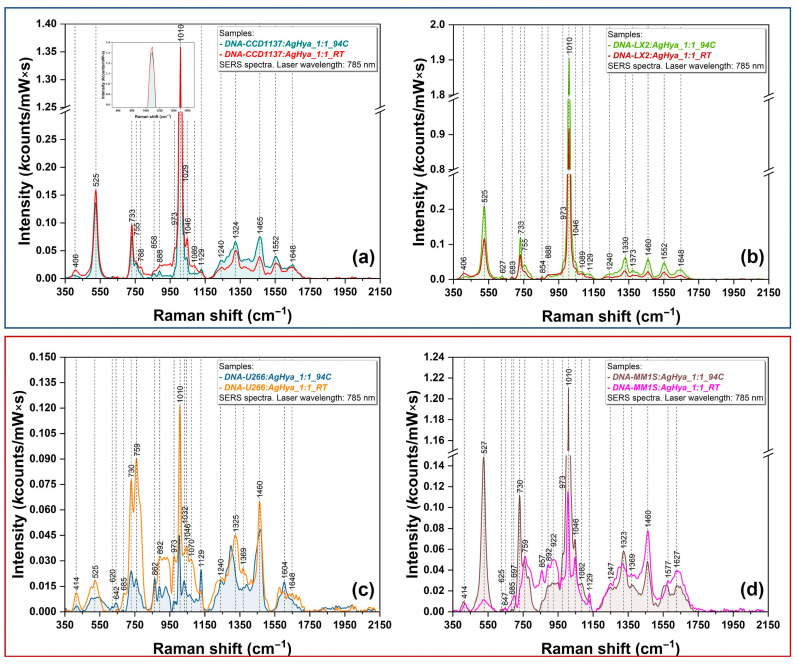
SERS spectra of DNA obtained from (**a**) CCD1137Sk (red—RT; jade—94 °C), (**b**) LX2 (scarlet red—RT; green—94 °C), (**c**) U266 (orange—RT; blue—94 °C), and (**d**) MM1S cells (viva magenta—RT; brown—94 °C). The spectra were recorded using a NIR (785 nm) excitation laser.

The SERS experiments were carried out before and after a heat treatment was performed at 94 °C, for 4 min, using silver plasmonic nanoparticles as substrates (their physical characterization is presented in Figures S1 and S2). These settings were also applied to silver nanoparticles, and the recorded spectra are presented in Figure S3. As can be seen, there are no differences between these measurements, allowing the conclusion that the heating step does not affect the nanoparticles. This heating treatment is frequently used in real-time (RT) PCR experiments for the denaturation of DNA templates. It has the role of separating the two DNA strands by thermally breaking the hydrogen bonds occurring between the complementary bases and making them available for primer annealing. This is the reason why we chose the same heating conditions for our SERS experiments. The SERS spectra recorded before (room temperature—RT) and after the heat treatment (94 °C) are presented in Figure 3 for all DNA samples isolated from normal (Figure 3a,b, upper blue rectangle) and cancer cell lines (Figure 3c,d, lower red rectangle).

The first observation that can be made is that in the case of normal DNA samples (CCD1137&LX2), the heating treatment has no effect on DNA analytes, the spectra being almost identical, both for band positions and band intensities.

A different behavior was encountered in the case of DNA extracted from cancer cells (Figure 3c,d). For these two samples, a clear distinction between the spectra recorded before and after the heat treatment can be observed. In the case of MM1S DNA, the treatment generated a SERS spectrum (brown spectrum in Figure 3d) very similar to the one recorded in the case of normal DNA. A huge increase in the intensity of the 527 and 730 cm^−1^ bands was observed after the heating. The most intense peak is 1010 cm^−1^. The intensity is also influenced by the treatment. A comparison of peaks’ intensities shows that the SERS spectrum of MM1S DNA recorded after the treatment is very similar to those recorded for “normal” DNA (Figure 3a,b), with 1010, 527/525, and 733/730 being the three most intense vibrational bands observed in these spectra.

In the case of U266 DNA, the spectra recorded before and after the treatment are the most different with respect to all four DNA samples. The vast majority of the vibrational bands recorded for the other DNA samples included in this study are still present but, in this case, the heat treatment induced a visible decrease in the intensity of all vibrational bands. In addition, in Figure S4, we represent all SERS spectra recorded on this sample in both conditions, namely at room temperature and after thermal treatment, proving the reproducibility of the SERS method. As further proof of experimental reproducibility, in Figure S5, we represent the 2D SERS mapping performed on the dried mixture of AgNPs and U266 DNA samples. The heat map superimposed on the optical image of the dried mixture was constructed using the intensity of the 1460 cm^−1^ vibrational band. The positions where the individual spectra were recorded are also marked in the figure. The mean spectrum, obtained using these individual spectra, is also highlighted in the figure.

The DNA methylation assay (Table 1) indicated that for this cell line, the concentration of 5 mC is the lowest.

**Figure 4:**
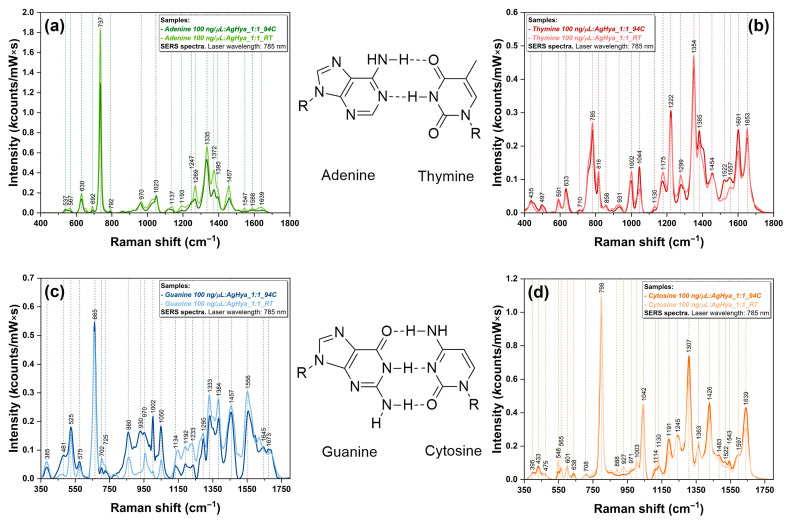
SERS spectra of DNA bases: (**a**) adenine (green); (**b**) thymine (red); (**c**) guanine (blue); (**d**) cytosine (orange).

The SERS spectra of the four DNA bases are presented in Figure 4. In order to study the influence of the heat treatment on each individual base, the spectra were recorded before and after the treatment, in a very similar manner to that for the DNA samples. As it was observed in the case of Raman measurement, for A, C, and G, the spectra exhibit a very intense band associated with the breathing mode (737/798/665 cm^−1^) and several intense bands in the 1200–1700 cm^−1^ region.

The interaction of the bases with the plasmonic substrate induced a slight shift in the band positions for these breathing modes (<12 cm^−1^). The most important observation that can be made for A, T, and C is that the heat treatment has no influence on their adsorption geometry on the plasmonic substrates, since the spectra recorded before and after are almost identical. However, the treatment’s influence is the most visible in the case of G.

Table 2 presents a tentative assignment for the principal vibrational bands recorded on DNA samples using Raman/SERS analysis.

## 4. Discussion

In the context of B-cell development and maturation, hematopoietic stem cells undergo multiple differentiation stages in the hematogenous marrow and lymphoid organs. Throughout this process, dynamic changes in DNA methylation occur, with a gradual disappearance of non-CpG methylation and CpG methylation-related changes observed in the early stages. In multiple myeloma (MM), a cancer affecting plasma cells, several tumor suppressor genes (e.g., CDKN2A, CDKN2B, SOCS1, and SHP1) are hypermethylated in the CpG islands of their promoters, leading to gene translation inhibition [37]. These findings highlight the role of DNA methylation in programming gene expression for B-cell differentiation and maturation under physiological conditions.

Plasma-cell differentiation is associated with epigenetic reprogramming, including a global loss of DNA methylation. Aberrant DNA methylation changes are observed in almost all stages of MM. Generally, the transition from monoclonal gammopathy of undetermined significance to MM is characterized by global hypomethylation and specific gene hypermethylation. However, during the progression from MM to plasma-cell leukemia, there is genome remethylating along with significant changes in gene expression [38].

Over time, much research has focused on DNA spectral analysis by developing various sensing mechanisms especially for clinical purposes [39]. Some of these methods were inspired by the drawbacks of molecular biology techniques such as laborious work, the need for skilled staff, time-consuming processes, and high costs. The Raman/SERS technique was implemented as a cost-efficient alternative that can deliver accurate and fast results [40]. It has the capacity to provide molecular detection based on the affinity of a specific molecule to the plasmonic substrate. The sample preparation step is a very important aspect of SERS, being able to influence the quality of the results.

In this study, we show that DNA stability and DNA interaction with the plasmonic nanoparticles is of paramount importance for generating reproducible and high-quality SERS spectra. In the case of Raman measurements, all the samples (DNA isolated from cells and DNA bases) of 100 ng/µL concentration were poured on the aluminum foil and allowed to dry before Raman spectra recording. The Raman spectra show the presence of principal vibrational bands specific to DNA molecules and DNA bases. In particular, it can be remarked that the vibrational bands located at 786 (C ring breathing [35]), 1010 (5 mC [36,41]), 1094 (deoxyribose phosphate backbone [33,35]), 1249 (A, T [30,33]), 1318 (G [35]), 1333 (A [32]), 1374 (A, T, G [30,31,33]), 1484 (A, T, G, C [30,32,33,35]), and 1575 cm^−1^ (A, G [30,32,33,35]) are more intense in the case of MM1S DNA as compared to the other samples. On the other hand, the vibrational bands located at 810 (G [32]), 863, 887, and 916 cm^−1^ (deoxyribose phosphate backbone [33]) are more intense for CCD1137Sk DNA.

The 1094 cm^−1^ band is one of the most meaningful since it is assigned to localized stretching vibrations of the phosphate group (OPO) belonging to the deoxyribose backbone. Its intensity is almost the same in the case of all DNA samples included in this study, suggesting a similar aggregation tendency of the samples investigated by Raman analysis. This peak will “disappear” in the SERS spectra recorded on the same samples. However, the Raman analysis cannot give a precise distinction between cancerous and normal DNA.

SERS measurements were recorded using two distinct preparation methods that allowed the analysis of DNA and DNA bases and the study of their stability and interaction with the plasmonic substrate. Firstly, all DNA samples and DNA bases were mixed with silver colloidal nanoparticles, and these mixtures were incubated at room temperature for 1 h. Then, two different strategies were applied. In the first case, the mixtures were analyzed at room temperature, while in the second one, the samples were measured after an additional heating step was performed at 94 °C for 4 min. The settings of the heat treatment procedure were chosen in order to denature the dsDNA and to obtain single strands of DNA that can bind easily to the silver nanoparticle surfaces. In a previous study, our group successfully employed this strategy for assessing the methylation landscape in the case of DNA samples isolated from leukemia cell lines [41].

As can be observed in Figure 3a,b (blue rectangle), the normal DNA SERS spectra recorded before and after heat treatment are very similar. This represents strong experimental proof that the integrity of DNA samples is not affected by the treatment.

On the contrary, in the case of MM cell lines (Figure 3c,d, red rectangle), the spectra recorded before and after the treatment present major modifications. This major finding can be directly correlated to the different degrees of DNA stability of normal and cancerous DNA. In the case of normal DNA, the heat treatment has no effect on the SERS spectra, suggesting that the dsDNA structure is not denatured after the treatment. On the contrary, the irregular methylation landscape present in cancer cells may be responsible for the low stability of cancerous DNA as compared to normal DNA.

The experimentally observed modification of the SERS spectra recorded on cancerous DNA samples after the heat treatment is a direct consequence of a modified interaction between temperature-induced denatured DNA and the plasmonic nanoparticles. This can be explained by the distinct methylation pattern of cancerous DNA compared to normal DNA, as shown by the methylation assay results (Table 1). It has to be stated that methylation assay offers a global view of the methylation landscape, whereas SERS spectroscopy provides a local one, at the nanoscale level. Moreover, due to different adsorption geometries generated by the modification of the methylation landscape, it was shown that the cancerous DNA binding to silver nanoparticles varies depending on the DNA nature and the temperature conditions applied during sample preparation [42]. Such differences can be observed in the case of SERS spectra recorded on MM1S and U266 DNA (Figure 3c,d). Moreover, since the SERS spectra recorded on the DNA bases before and after the heat treatment (using the same experimental conditions as for DNA samples) showed minor differences (Figure 4), one can conclude that the differences observed for cancerous DNA samples can be attributed only to structural DNA instability generated by the hypomethylation process. The heat treatment induced major modifications of the cancerous DNA samples, whereas in the case of normal DNA, its structure was not affected by the heat treatment.

Differences in the intensity of the vibrational bands have been also observed. Cancerous DNA vibrational bands are weaker in intensity as compared to those of normal DNA due to their modified irregular structure that has a great impact on their interaction with the plasmonic nanoparticles. In the case of normal cells, DNA methylation is beneficial for gene expression regulation by enhancing genome stability [43]. On the other hand, cancerous DNA hypermethylation at specific loci affects the organization of the genome and contributes to its destabilization [44]. This molecular biology event of crucial importance in cancer development was experimentally proven in this study by means of SERS. This is the reason why the heat treatment induced spectral modifications only in the case of cancerous DNA samples. Since the SERS analysis performed on the four DNA bases under the same experimental conditions (Figure 4) showed no differences between the spectra recorded before and after the treatment, one can conclude that the spectral differences observed only in the case of cancerous DNA represent a direct consequence of their structural instability provoked by the presence of an irregular methylation landscape.

These results prove that Raman/SERS techniques allow the real-time analysis of subtle changes in DNA structures, including variations in methylation patterns and specific biomarkers associated with diseases such as cancer. By identifying subtle molecular changes, Raman/SERS may contribute to the early detection of diseases, enabling timely intervention and improved patient outcomes.

## 5. Conclusions

In this study, we have implemented innovative DNA sample preparation techniques for obtaining accurate SERS spectra that can be used to evaluate the molecular modifications associated with cancer together with DNA stability. Silver colloids were mixed with DNA isolated from normal and cancer cells and with pure DNA bases. The mixing technique allowed the interaction of DNA samples with silver nanoparticle surfaces. The spectra were recorded before and after a heat treatment was performed at 94 °C in order to evaluate the structural stability of the double-stranded DNA and to analyze the effect of the heat treatment on the adsorption geometry of DNA samples on silver nanoparticle surfaces.

SERS results have shown significant differences between temperature-conditioned samples only in the case of DNA extracted from MM cell lines. By considering epigenetics, which plays a crucial role in cancer cell development (especially the methylation of C), experimentally proving DNA stability and its interaction with nanomaterials may offer new perspectives in studying genome instability. The SERS analysis included in this study was able to clearly discriminate cancerous DNA from normal DNA.

Further studies need to be performed in order to complete the knowledge about this topic of ultimate importance. The integration of nanotechnology and vibrational spectroscopy not only advances our understanding of these interactions but also opens new avenues for the design of efficient and sensitive biosensors. The study underscores the importance of comprehending DNA–nanoparticle interactions, laying a foundation for further exploration and their potential application in the field of biomedicine, particularly in early disease detection and personalized medicine.

## Figures and Tables

**Table 1:** DNA methylation pattern.

Cell Line	5 mC (%) (5 mC/Total DNA)
MM1S	0.268
U266	0.160
LX2	0.759
CCD1137Sk	0.701

**Table 2:** Tentative assignment of Raman and SERS bands for DNA samples.

Raman Wavenumber (cm^−1^)	SERS Wavenumber (cm^−1^)	Assignments	References
	406	C	[30]
420	414	T	[30,31,32]
482		T	[30]
502		T, G	[32,33]
535		A	[30,32,33]
582		T, G	[32]
598		C	[30,32,33]
	620–627	C, T, A	[30,32,33]
632		A, C	[33]
	642–645	G	[30]
670		G, C	[32,33,34,35]
	683–685	G, C	[32,33,34,35]
	697	C, deoxyribose phosphate backbone	[32,33]
727	730–733	A	[30,32,33,34,35]
762	755–759	T	[30]
786	788	T, C	[31,33,35]
810		T, G	[30,32]
863	854–862	T, G	[30,31,32]
887	888–892	Deoxyribose phosphate backbone	[33]
916	922	Deoxyribose phosphate backbone	[33]
	973	C, deoxyribose phosphate backbone	[30,32,33]
991		C, deoxyribose phosphate backbone	[30,33]
1009	1010	T, deoxyribose phosphate backbone, 5-mC	[31,33,36]
	1029–1032	A, G, deoxyribose phosphate backbone	[32,33]
	1046	T, G	[30,31]
1094	1082–1089	Deoxyribose phosphate backbone	[33,35]
	1129	A	[30,32]
1182		T, C	[32,33]
1249	1240–1247	A, T	[30,33]
1318	1323–1325	G	[33]
1333	1330	A	[30,32,33,35]
1374	1369–1373	A, T, G	[30,31,33]
1420		T, C, G	[30,31,32,33]
	1460–1465	A, T, C, G, deoxyribose phosphate backbone	[30,33]
1484		A, T, C, G	[32,33,35]
1530		C	[30,32,33]
	1552	G	[30]
1575	1577	A, G	[32,33,34,35]
1601	1604	C, G	[30,32,33]
	1627	T	[33]
	1648	T, C	[30,33,34]
1670		T, G	[30,31,32,33]

## Data Availability

Data are contained within the article and Supplementary Materials.

## Supplementary Materials

The following supporting information can be downloaded at https://www.mdpi.com/article/10.3390/bios14010041/s1: Table S1: Mycoplasma assay results; Figure S1: Absorbance spectra of silver nanoparticles before tangential flow filtration (pink spectrum) and after filtration (orange spectrum); Figure S2: Transmission electron microscopy image of filtered silver nanoparticles (a). Size distribution graph of filtered silver nanoparticles (b); Figure S3: SER spectra of silver nanoparticles at room temperature (pink spectrum) versus the nanoparticles subjected to a heating step at 94 °C, for 4 min (blue spectrum); Figure S4: SER spectra of U266 cell DNA at room temperature (a) and 94 °C (b). Both red and blue spectra represent the mean of 2 spectral maps of 50 acquisitions. Figure S5: 2D SERS analysis of the dried mixture composed of AgNPs and U266 DNA samples. The heat map constructed using the intensity of 1460 cm^−1^ vibrational band is superposed over the optical image of the dried mixture (left). The exact positions where the individual spectra were recorded are marked in the left inset by black points. All the individual spectra (grey) together with the mean spectrum (yellow) are also included in the figure (right).

## References

[1] Globocan Cancer Observatory. https://Gco.Iarc.Fr/.

[2] Sung H., Ferlay J., Siegel R.L., Laversanne M., Soerjomataram I., Jemal A., Bray F. (2021). Global Cancer Statistics 2020: GLOBOCAN Estimates of Incidence and Mortality Worldwide for 36 Cancers in 185 Countries. CA Cancer J. Clin..

[3] Cottini F., Hideshima T., Suzuki R., Tai Y.-T., Bianchini G., Richardson P.G., Anderson K.C., Tonon G. (2015). Synthetic Lethal Approaches Exploiting DNA Damage in Aggressive Myeloma. Cancer Discov..

[4] Botrugno O.A., Tonon G. (2021). Genomic Instability and Replicative Stress in Multiple Myeloma: The Final Curtain?. Cancers.

[5] Baylin S.B., Jones P.A. (2016). Epigenetic Determinants of Cancer. Cold Spring Harb. Perspect. Biol..

[6] Banerjee H.N., Verma M. (2009). Epigenetic Mechanisms in Cancer. Biomark. Med..

[7] Ors Kumoglu G., Sendemir A., Tanyolac M.B., Bilir B., Kucuk O., Missirlis Y.F. (2022). Epigenetic Mechanisms in Cancer. Longhua Chin. Med..

[8] Holliday R., Grigg G.W. (1993). DNA Methylation and Mutation. Mutat. Res. Fundam. Mol. Mech. Mutagen..

[9] Das P.M., Singal R. (2004). DNA Methylation and Cancer. J. Clin. Oncol..

[10] Baylin S.B. (2001). Aberrant Patterns of DNA Methylation, Chromatin Formation and Gene Expression in Cancer. Hum. Mol. Genet..

[11] Feinberg A.P., Vogelstein B. (1983). Hypomethylation Distinguishes Genes of Some Human Cancers from Their Normal Counterparts. Nature.

[12] Baylin S.B., Ohm J.E. (2006). Epigenetic Gene Silencing in Cancer—A Mechanism for Early Oncogenic Pathway Addiction?. Nat. Rev. Cancer.

[13] Felsher D.W., Bishop J.M. (1999). Transient Excess of *MYC* Activity Can Elicit Genomic Instability and Tumorigenesis. Proc. Natl. Acad. Sci. USA.

[14] Denko N.C., Giaccia A.J., Stringer J.R., Stambrook P.J. (1994). The Human Ha-Ras Oncogene Induces Genomic Instability in Murine Fibroblasts within One Cell Cycle. Proc. Natl. Acad. Sci. USA.

[15] Jin B., Robertson K.D. (2013). DNA Methyltransferases, DNA Damage Repair, and Cancer. Epigenetic Alterations in Oncogenesis.

[16] Saitoh T., Oda T. (2021). DNA Damage Response in Multiple Myeloma: The Role of the Tumor Microenvironment. Cancers.

[17] Petrilla C., Galloway J., Kudalkar R., Ismael A., Cottini F. (2023). Understanding DNA Damage Response and DNA Repair in Multiple Myeloma. Cancers.

[18] Mereu E., Abbo D., Paradzik T., Cumerlato M., Bandini C., Labrador M., Maccagno M., Ronchetti D., Manicardi V., Neri A. (2023). Euchromatic Histone Lysine Methyltransferase 2 Inhibition Enhances Carfilzomib Sensitivity and Overcomes Drug Resistance in Multiple Myeloma Cell Lines. Cancers.

[19] Stiufiuc R., Iacovita C., Lucaciu C.M., Stiufiuc G., Dutu A.G., Braescu C., Leopold N. (2013). SERS-Active Silver Colloids Prepared by Reduction of Silver Nitrate with Short-Chain Polyethylene Glycol. Nanoscale Res. Lett..

[20] Nie S., Emory S.R. (1997). Probing Single Molecules and Single Nanoparticles by Surface-Enhanced Raman Scattering. Science.

[21] Kneipp K., Wang Y., Kneipp H., Perelman L.T., Itzkan I., Dasari R.R., Feld M.S. (1997). Single Molecule Detection Using Surface-Enhanced Raman Scattering (SERS). Phys. Rev. Lett..

[22] Vo-Dinh T. (1998). Surface-Enhanced Raman Spectroscopy Using Metallic Nanostructures. TrAC Trends Anal. Chem..

[23] Borșa R.-M., Toma V., Onaciu A., Moldovan C.-S., Mărginean R., Cenariu D., Știufiuc G.-F., Dinu C.-M., Bran S., Opriș H.-O. (2023). Developing New Diagnostic Tools Based on SERS Analysis of Filtered Salivary Samples for Oral Cancer Detection. Int. J. Mol. Sci..

[24] Faur C.I., Dinu C., Toma V., Jurj A., Mărginean R., Onaciu A., Roman R.C., Culic C., Chirilă M., Rotar H. (2023). A New Detection Method of Oral and Oropharyngeal Squamous Cell Carcinoma Based on Multivariate Analysis of Surface Enhanced Raman Spectra of Salivary Exosomes. J. Pers. Med..

[25] Munteanu V.C., Munteanu R.A., Gulei D., Mărginean R., Schițcu V.H., Onaciu A., Toma V., Știufiuc G.F., Coman I., Știufiuc R.I. (2022). New Insights into the Multivariate Analysis of SER Spectra Collected on Blood Samples for Prostate Cancer Detection: Towards a Better Understanding of the Role Played by Different Biomolecules on Cancer Screening: A Preliminary Study. Cancers.

[26] Leopold N., Lendl B. (2003). A New Method for Fast Preparation of Highly Surface-Enhanced Raman Scattering (SERS) Active Silver Colloids at Room Temperature by Reduction of Silver Nitrate with Hydroxylamine Hydrochloride. J. Phys. Chem. B.

[27] Moisoiu V., Socaciu A., Stefancu A., Iancu S., Boros I., Alecsa C., Rachieriu C., Chiorean A., Eniu D., Leopold N. (2019). Breast Cancer Diagnosis by Surface-Enhanced Raman Scattering (SERS) of Urine. Appl. Sci..

[28] Știufiuc G.F., Toma V., Buse M., Mărginean R., Morar-Bolba G., Culic B., Tetean R., Leopold N., Pavel I., Lucaciu C.M. (2020). Solid Plasmonic Substrates for Breast Cancer Detection by Means of SERS Analysis of Blood Plasma. Nanomaterials.

[29] Safar W., Azziz A., Edely M., Lamy de la Chapelle M. (2023). Conventional Raman, SERS and TERS Studies of DNA Compounds. Chemosensors.

[30] De Gelder J., De Gussem K., Vandenabeele P., Moens L. (2007). Reference Database of Raman Spectra of Biological Molecules. J. Raman Spectrosc..

[31] Aroca R., Bujalski R. (1999). Surface Enhanced Vibrational Spectra of Thymine. Vib. Spectrosc..

[32] Otto C., van den Tweel T.J.J., de Mul F.F.M., Greve J. (1986). Surface-enhanced Raman Spectroscopy of DNA Bases. J. Raman Spectrosc..

[33] Prescott B., Steinmetz W., Thomas G.J. (1984). Characterization of DNA Structures by Laser Raman Spectroscopy. Biopolymers.

[34] Garcia-Rico E., Alvarez-Puebla R.A., Guerrini L. (2018). Direct Surface-Enhanced Raman Scattering (SERS) Spectroscopy of Nucleic Acids: From Fundamental Studies to Real-Life Applications. Chem. Soc. Rev..

[35] Barhoumi A., Zhang D., Tam F., Halas N.J. (2008). Surface-Enhanced Raman Spectroscopy of DNA. J. Am. Chem. Soc..

[36] Moisoiu V., Stefancu A., Iancu S.D., Moisoiu T., Loga L., Dican L., Alecsa C.D., Boros I., Jurj A., Dima D. (2019). SERS Assessment of the Cancer-Specific Methylation Pattern of Genomic DNA: Towards the Detection of Acute Myeloid Leukemia in Patients Undergoing Hematopoietic Stem Cell Transplantation. Anal. Bioanal. Chem..

[37] Kulis M., Merkel A., Heath S., Queirós A.C., Schuyler R.P., Castellano G., Beekman R., Raineri E., Esteve A., Clot G. (2015). Whole-Genome Fingerprint of the DNA Methylome during Human B Cell Differentiation. Nat. Genet..

[38] Walker B.A., Wardell C.P., Chiecchio L., Smith E.M., Boyd K.D., Neri A., Davies F.E., Ross F.M., Morgan G.J. (2011). Aberrant Global Methylation Patterns Affect the Molecular Pathogenesis and Prognosis of Multiple Myeloma. Blood.

[39] Pyrak E., Krajczewski J., Kowalik A., Kudelski A., Jaworska A. (2019). Surface Enhanced Raman Spectroscopy for DNA Biosensors—How Far Are We?. Molecules.

[40] Chen C., Liu W., Tian S., Hong T. (2019). Novel Surface-Enhanced Raman Spectroscopy Techniques for DNA, Protein and Drug Detection. Sensors.

[41] Onaciu A., Toma V., Moldovan C., Țigu A.B., Cenariu D., Culic C., Borșa R.M., David L., Știufiuc G.F., Tetean R. (2022). Nanoscale Investigation of DNA Demethylation in Leukemia Cells by Means of Ultrasensitive Vibrational Spectroscopy. Sensors.

[42] Sheaffer K.L., Elliott E.N., Kaestner K.H. (2016). DNA Hypomethylation Contributes to Genomic Instability and Intestinal Cancer Initiation. Cancer Prev. Res..

[43] Zhou D., Robertson K.D. (2016). Role of DNA Methylation in Genome Stability. Genome Stability.

[44] Madakashira B.P., Sadler K.C. (2017). DNA Methylation, Nuclear Organization, and Cancer. Front. Genet..

