# Supplementary material for: Understanding DNA Epigenetics by Means of Raman/SERS Analysis for Cancer Detection
Source: Biosensors (Basel). 2024 Jan 12;14(1):41. doi: 10.3390/bios14010041 (PMC10813173; doi:10.3390/bios14010041)
Supplement: Supplementary file 1 [file biosensors-14-00041-s001.zip › biosensors-2773407-supplementary.pdf]

Supplementary File

Understanding DNA Epigenetics by Means of Raman/SERS Analysis for Cancer Detection

Luca David [1], Anca Onaciu [2,3], Valentin Toma [2], Rareș-Mario Borșa[2,4], Cristian Moldovan [2,3], Adrian-Bogdan Țigu [2], Diana Cenariu [2], Ioan Șimon[5], Gabriela-Fabiola Știufiuc [6], Eugen Carasevici [7], Brîndușa Drăgoi [7], Ciprian Tomuleasa [2,8,9], Rareș-Ionuț Știufiuc [2,3,7,*]

1 Faculty of Medicine, "Iuliu Hatieganu" University of Medicine and Pharmacy, 400349 Cluj-Napoca, Romania; david.luca@elearn.umfcluj.ro
2 MedFuture—Research Center for Advanced Medicine, "Iuliu Hatieganu" University of Medicine and Pharmacy, 400349 Cluj-Napoca, Romania; anca.onaciu@umfcluj.ro (A.O.); valentin.toma@umfcluj.ro (V.T.); rares.mari.borsa@elearn.umfcluj.ro (R.-M.B.); moldovan.cristian@umfcluj.ro (C.M.); bogdan.tigu@umfcluj.ro (A.-B.Ț.); diana.cenariu@umfcluj.ro (D.C.); ciprian.tomuleasa@umfcluj.ro (C.T.)
3 Department of Pharmaceutical Physics & Biophysics, "Iuliu Hatieganu" University of Medicine and Pharmacy, 400349 Cluj-Napoca, Romania
4 Department of Maxillofacial Surgery and Implantology, "Iuliu Hațieganu" University of Medicine and Pharmacy, 400349 Cluj-Napoca, Romania
5 Department of Surgery, "Iuliu Hatieganu" University of Medicine and Pharmacy, 400349 Cluj-Napoca, Romania; ioan.simon@umfcluj.ro
6 Faculty of Physics, "Babes Bolyai" University, 400084 Cluj-Napoca, Romania; gabriela.stiufiuc@ubbcluj.ro
7 Nanotechnology Laboratory, TRANSCEND Research Center, Regional Institute of Oncology, 700483 Iasi, Romania; eugen.carasevici@gmail.com (E.C.); transcendbd@iroiasi.ro (B.D.)
8 Department of Hematology, "Iuliu Hatieganu" University of Medicine and Pharmacy, 400349 Cluj-Napoca, Romania
9 Department of Hematology, "Ion Chiricuta" Clinical Cancer Center, 400015 Cluj-Napoca, Romania
* Correspondence: rares.stiufiuc@umfcluj.ro; Tel.: +40-726-340-278

Table S1. Mycoplasma assay results

	LX2	CCD1137Sk	U266	MM1S	PBS
Read A	107	98	110	155	67
Read B	75	68	67	102	37
	0.70	0.69	0.61	0.66	0.55

To be noticed:

- Less than 1 for negative samples (Mycoplasma negative)
- 1-1.2 for samples that need reevaluation (Borderline)
- Over 1.2 for positive samples (Mycoplasma positive)

The negative control was sterile PBS.

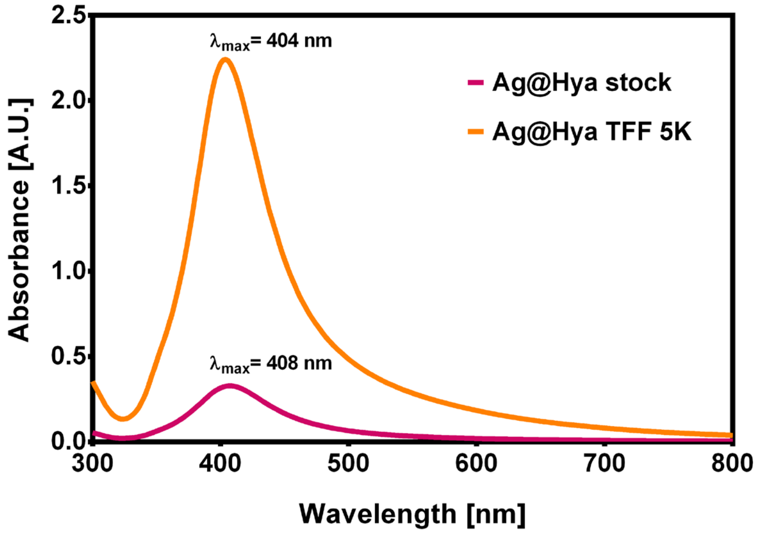

Figure S1. Absorbance spectra of silver nanoparticles before tangential flow filtration (pink spectrum) and after filtration (orange spectrum)

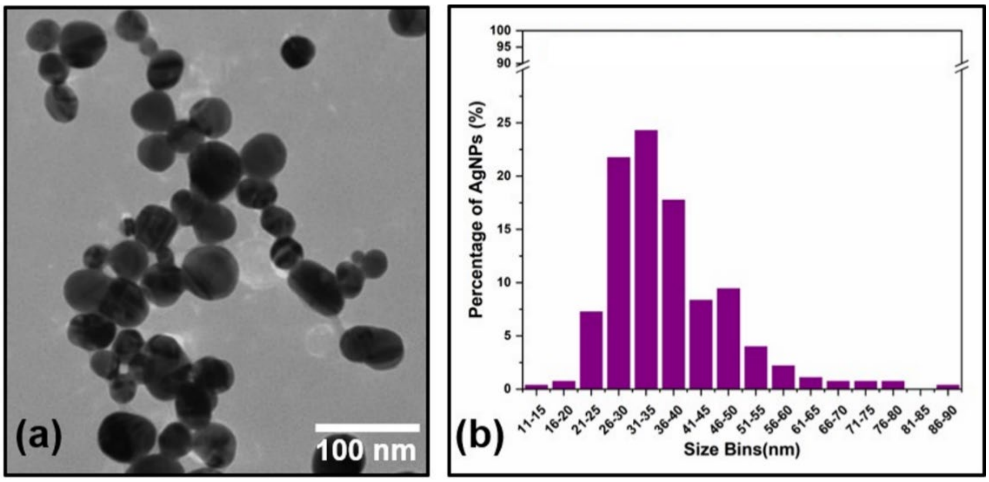

Figure S2. Transmission electron microscopy image of filtered silver nanoparticles (a). Size distribution graph of filtered silver nanoparticles (b).

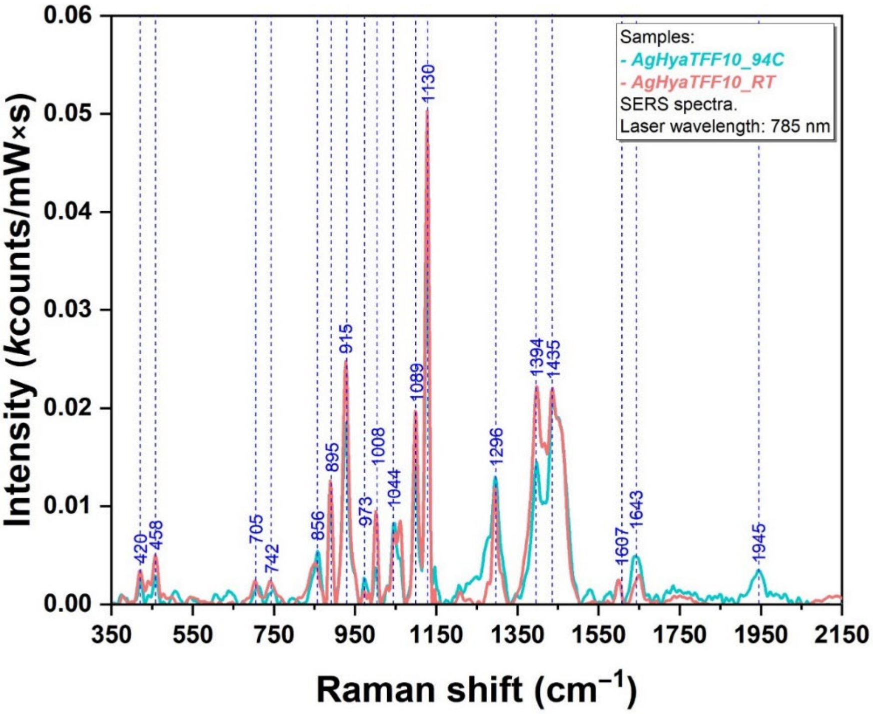

Figure S3. SERS spectra of silver nanoparticles employed as plasmonic substrates at room temperature (pink spectrum) versus the nanoparticles subjected to a heating step at 94°C, for 4 minutes (blue spectrum).

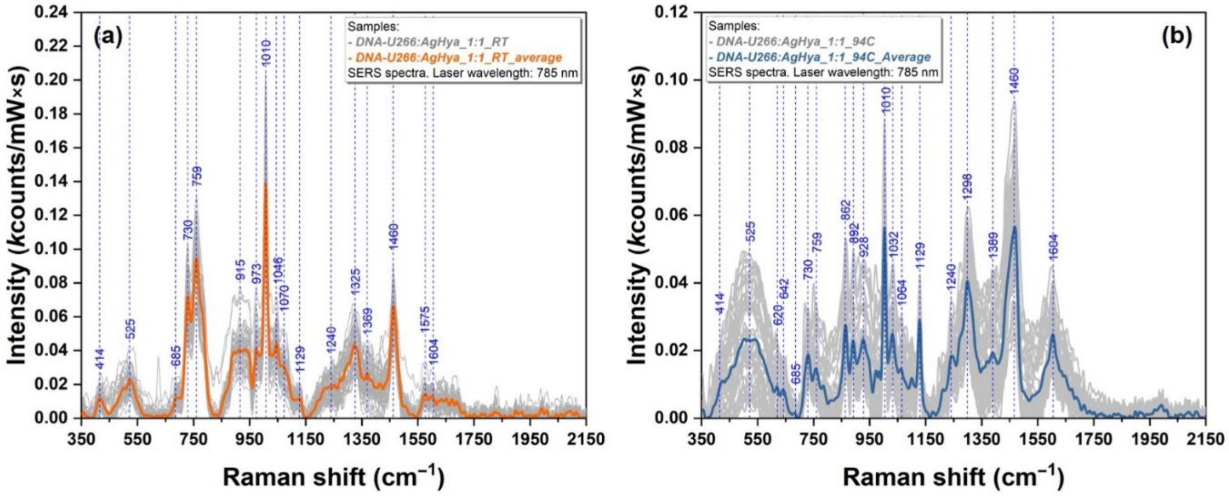

Figure S4. Individual and mean SERS spectra of U266 cells DNA at room temperature (a) and 94°C (b). Both orange and blue spectra represent the mean of 2 spectral maps of 50 acquisitions.

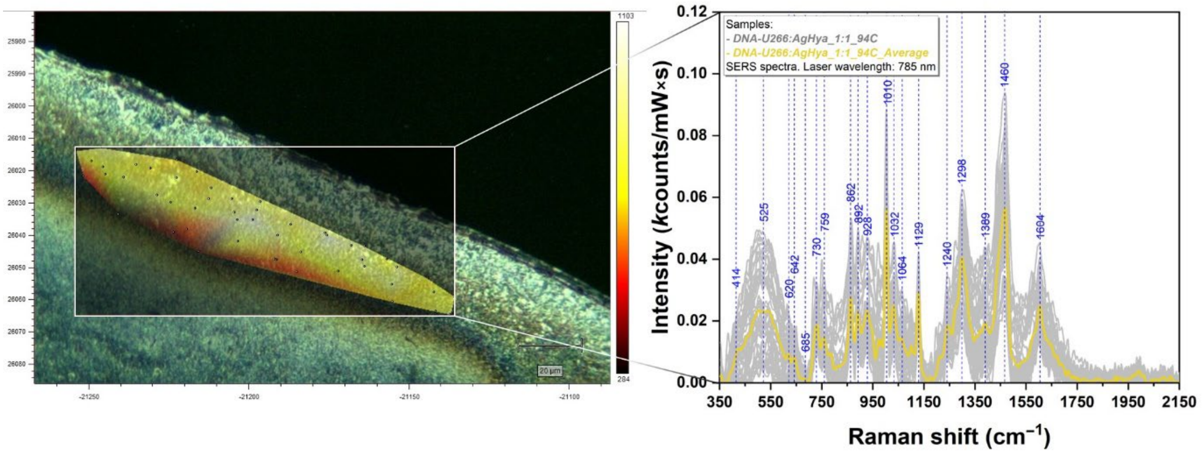

Figure S5. 2D SERS analysis of the dried mixture composed of AgNPs and U266 DNA samples. The heat map constructed using the intensity of 1460 cm-1 vibrational band is superposed over the optical image of the dried mixture (left). The exact positions where the individual spectra were recorded are marked in the left inset by black points. All the individual spectra (grey) together with the mean spectrum (yellow) are also included in the figure (right).
